# METTL16 Inhibits the Malignant Progression of Epithelial Ovarian Cancer through the lncRNA MALAT1/*β*-Catenin Axis

**DOI:** 10.1155/2023/9952234

**Published:** 2023-10-28

**Authors:** Changshu Li, Ji Liu, Yuanyuan Lyu, Shizhang Ling, Yonghong Luo

**Affiliations:** ^1^Department of Obstetrics and Gynecology, The First Affiliated Hospital of Wannan Medical College (Yijishan Hospital of Wannan Medical College), Wuhu 241001, Anhui, China; ^2^The Translational Research Institute for Neurological Disorders, Wuhu 241001, Anhui, China; ^3^Interdisciplinary Research Center of Neuromedicine and Chemical Biology of Wannan Medical College and Anhui Normal University, Wuhu, Anhui 241001, China; ^4^Department of Neurosurgery, The First Affiliated Hospital of Wannan Medical College (Yijishan Hospital of Wannan Medical College), Wuhu, Anhui 241001, China

## Abstract

Epithelial ovarian cancer (EOC) ranks third in the incidence of gynecological malignancies. m6A methylation as RNA modification plays a crucial role in the evolution, migration, and invasion of various tumors. However, the role of m6A methylation in ovarian cancer (OC) only recently has begun to be appreciated. Therefore, we used various bioinformatic methods to screen the public GEO datasets of epithelial ovarian cancer (EOC) for m6A methylation-related regulators. We identified methyltransferase 16 (METTL16) that was dramatically downregulated in EOC as such a regulator. We also identified metastasis-associated lung adenocarcinoma transcript 1 (MALAT1), a known target lncRNA of METTL16, in these five GEO datasets. RT-qPCR and immunohistochemical staining confirmed that compared with the normal ovarian tissues and cells, METTL16 was significantly downregulated, while lncRNA MALAT1 was significantly upregulated, in 30 EOC tissues of our own validation cohorts and EOC cell lines, revealing a negative correlation between METTL16 and lncRNA MALAT1. Moreover, our analysis unveiled a correlation between downregulated METTL16 and the known adverse prognostic factors of EOC patients in our own cohorts. The CCK-8, EdU, scratch wound healing, and transwell invasion assays revealed that METTL16 significantly suppressed the proliferating, migrating, and invading abilities of OC cells. The inhibitory effects of METTL16 on the in vivo tumor growth of EOC cells were measured by subcutaneous tumor formation assay in mice. Furthermore, the RIP, RNA stability assay, western blotting, and cytoimmunofluorescence staining showed that METTL16 hindered the growth of EOC cells through promoting the degradation of MALAT1 by binding that, in turn, upregulates *β*-catenin protein and promotes nuclear transport of *β*-catenin protein in EOC cells. This study suggests that METTL16 acts as a tumor suppressor gene of EOC by achieving its inhibitory function on the malignant progression of EOC through the METTL16/MALAT1/*β*-catenin axis that are new targets for EOC diagnosis and therapy.

## 1. Introduction

Among gynecologic malignancies, ovarian cancer (OC) exhibits the highest fatality rate [1]. Many factors, such as inconspicuous early symptoms, easy metastasis and recurrence, and the lack of effective long-term chemotherapy, are closely associated with this outcome [2]. Epithelial ovarian cancer (EOC) is the most aggressive form of ovarian malignancies and exhibits a high propensity for abdominal metastasis, frequently resulting in the development of severe ascites and intestinal obstruction, ultimately leading to patient fatality. The International Federation of Gynecology and Obstetrics data show that the majority of EOC patients are already at stages III–IV when diagnosed, with a 5-year survival rate below 30% [3]. Extensive genetic and epigenetic modifications play a pivotal role in the malignant course of EOC. Therefore, further exploration of genes involved in epigenetic modification in EOC will provide valuable insights for identifying potential therapeutic targets and thus effectively improving the prognosis.

N6-methyladenosine (m6A) is the most common epigenetic modification found in mammalian RNAs, exerting a crucial function in the biological regulation of RNA activity [4]. Many m6A methylases have recently been found to be associated with the malignant progression of OC [5]. For example, Nie et al. [6] discovered that the expression of ALKBH5 was elevated in cisplatin-resistant EOC and exhibited a substantial correlation with the resistance of EOC cells to cisplatin, both in vivo and in vitro. Wang et al. [7] found that WTAP might be an ideal prognostic biomarker or therapeutic target in OC. Li et al. [8] developed a risk score model based on three genes (VIRMA, IGF2BP1, and HNRNPA2B1) related to m6A modification as potential prognostic biomarkers for OC. Recent evidence also suggested that FTO and YTHDC2 were significantly associated with OC grade, while ALKBH5, METTL3, METTL14, RBM15, and YTHDF1 were also significantly associated with the OC stage [5]. Bi et al. [9] demonstrated that METTL3 promoted the development as well as the metastasis of OC through enhancing the maturation of pri-miR-1246 and inhibiting CCNG2 expression. All these studies show that m6A methylases and m6A modification of the target RNAs by these methylases are involved in OC.

Human methyltransferase-like protein (METTL) acts as a “writer” of the m6A modification and adds the m6A modification to specific RNAs. METTL3 and METTL14 have been found to modulate the activity of some oncogenes, thus promoting the progression of a variety of cancers [10, 11]. Recently, METTL16 was also identified as an m6A methyltransferase, but its functional studies have only been limited to the addition of m6A to MAT2A mRNA and several classes of noncoding RNAs, such as U6 snRNA, MALAT1, and XIST [12–14]. METTL16 is essential in the early stages of mouse embryo development [15], but its biological function is still not clear. Several reports have also implicated METTL16 in certain types of cancers [16–19]. In colorectal cancer, mutations in key residues of METTL16, such as R200Q or G110C, were found, suggesting the association of METTL16 with the disease [16]. In patients with soft tissue sarcoma, the copy number variations of the METTL16 gene are closely connected to the overall survival [17]. Downregulation of METTL16 has recently been related to cancer progression and poor overall survival in patients with hepatocellular carcinoma and tumors of the endocrine system [18, 19]. Although it is known that METTL16 is involved in several types of cancers mentioned above, its biological function in OC is not clear.

This study aimed to determine the biological function as well as the downstream molecular mechanism of METTL16 in OC. First, we identified METTL16 as a differentially expressed gene (DEG) in EOC using the public GEO datasets and predicted lncRNA MALAT1 as a potential downstream target of METTL16. Then, we observed an inverse relationship between the expression levels of METTL16 and lncRNA MALAT1 in the EOC tissues and cells of our cohorts. Further mechanistic studies showed that METTL16 inhibited the progression of EOC by binding to MALAT1 through the lncRNA MALAT1/*β*-catenin axis. These discoveries will provide novel targets for EOC diagnosis and treatment, thereby potentially advancing EOC treatment strategies and improving the prognosis of EOC patients.

## 2. Materials and Methods

### 2.1. Patient Samples

Thirty tissues of EOC, as well as their adjacent noncancerous tissues, were obtained from the patients who received cytoreductive surgery from February 2020 to February 2022 at the First Affiliated Hospital of Wannan Medical College. The inclusion criteria for all patients in this study were that the patients met the diagnostic criteria for OC, had complete background data and never received chemotherapy and radiotherapy before surgery, and all underwent initial optimal tumor resection, as well as the postoperative specimens were pathologically confirmed as primary EOC or adjacent noncancerous tissues. Patients with any of the subsequent conditions were excluded from this study: other malignancies, severe blood-borne diseases, severe heart, liver, kidney, and lung dysfunctions, immunodeficiency, coagulation dysfunction, diabetes, or other chronic metabolic disorders. Based on the standard schedules and doses, the postoperative adjuvant therapies were administered. Staining of the paraffin sections of all the collected samples was performed at the Department of Pathology, the First Affiliated Hospital of Wannan Medical College. The follow-up data of all these enrolled patients in this study were also collected. The study protocol was approved by the Ethics Committee of the First Affiliated Hospital of Wannan Medical College.

### 2.2. Cell Lines, Cell Culture, and Transfection

Five OC cell lines, namely OVCAR3, SKOV3, HO8910, ES-2, and A2780, as well as the human epithelial ovarian cell line (IOSE80), were procured from the Chinese Academy of Sciences. Human A2780, SKOV3, and OVCAR3 OC cells were cultured in RPMI 1640 medium (Gibco, Beijing, China) supplemented with 10% fetal bovine serum (FBS) and 1% penicillin/streptomycin antibiotics. Cells in passage 3 were used in all experiments. The small interfering RNAs (si-RNAs) (for transient knockdown), sh-RNA constructs (for stable knockdown), and overexpression constructs utilized for transfection were custom-designed and produced by GenePharma (Shanghai, China). The stable knockdown construct of sh-METTL16 was obtained by subcloning one of siMETTL16 sequences into a lentiviral vector, LV3, from GenePharma (Shanghai, China). Subsequently, SKOV3 and OVCAR3 cells were transfected with sh-METTL16, followed by the application of 2 *μ*g/mL puromycin to select stable METTL16-deficient cells. A2780 cells, on the other hand, were transfected with an overexpressing plasmid for METTL16 (oe-METTL16). To silence the expression of MALAT1, specific si-RNA was used in SKOV3 and OVCAR3 cells. The transfection of lentiviral vectors, si-RNAs, and sh-RNA constructs was carried out using GP-transfect-Mate (GenePharma).

### 2.3. Immunohistochemical Staining (IHC)

The tissue sections were deparaffinized in an oven for 25 min at 60°C, followed by the incubation in xylene solution for an additional 20 min. Next, a series of decreasing concentrations of ethanol solutions (100%, 95%, 80%, and 60%) were used for the rehydration of the tissue sections. Then, the slides were covered with target retrieval solution and heated in a microwave oven at medium power for 15 min. To quench the activity of endogenous peroxidase in tissue sections, the slides were incubated with a solution of 3% H_2_O_2_ for 15 min at room temperature. The peroxidase method was conducted for the staining of all tissue samples. In short, following an overnight incubation at 4°C with primary rabbit anti-METTL16 antibody (19924-1-AP, diluted 1 : 500, Proteintech), the tissue sections were subsequently incubated with HRP-conjugated secondary antibody (A0208, Beyotime Biotechnology) for 40 min at room temperature. Visualization was achieved by using diaminobenzidine for a duration of 3 min. Subsequently, they were immersed in a hematoxylin bath for nuclei staining for 2.5 min at room temperature. All the tissue sections were evaluated under a light microscope, and five fields of view were randomly chosen for photography at magnifications of 200x and 400x. Brownish-yellow granular staining in tumor nuclei and cytoplasm was deemed as positive staining. The tissue sections were assessed based on the intensity of staining (0–3 for the absence of staining, faint yellow, light brown, and dark brown staining, respectively) and the proportion of positive staining (0%–3% for 0%–25%, 26%–50%, 51%–75%, and 76%–100%, respectively). An *H*-score was obtained by combining the degree of staining and the extent of positivity. A total *H*-score ≤ 3 and ≧4 was defined as low- and high-expression levels of METTL16, respectively. The IHC results were evaluated separately by two experienced pathologists who were unaware of the groupings. In case of any disagreements in terms of *H*-scoring, the third pathologist was consulted for his or her input.

### 2.4. Quantitative Real-Time PCR (RT-qPCR)

The TRIzol reagent (TransGen Biotech, Beijing, China) was employed to isolate total RNA from the indicated samples. The synthesis of first-strand cDNA was performed using a reverse transcription kit (TransGen Biotech, Beijing, China). The PCR reaction was carried out by Bestar® SYBR Green qPCR Master Mix on an ABI 7500 PCR system (Applied Biosystems Inc.). The steps for the PCR reactions were as follows: initial denaturation at 95°C for 5 min, followed by 40 cycles of amplification at 95°C for 5 s, 60°C for 10 s, and 72°C for 15 s. The expression level of the GAPDH gene was used as an internal control. Relative expression levels were calculated according to Formula 2^−*ΔΔ*Ct^. The results of RT-qPCR were further statistically analyzed and graphed using GraphPad Prism 7.0 (GraphPad Software, USA). Primer sequences were as follows: METTL16 (forward, 5′-ACAGAAGACACTCCTGATGG-3′, reverse, 5′-TTAACAGAACTAGGCGGAGG-3′), MALAT1 (forward, 5′-GCTCTGTGGTGTGGGATTGA-3′, reverse, 5′-GTGGCAAAATGGCGGACTTT-3′), and GAPDH (forward, 5′-AGCCTCAAGATCATCAGCAATG-3′, reverse, 5′-ATGGACTGTGGTCATGAGTCCTT-3′).

### 2.5. Protein Extraction and Western Blotting

For the isolation of the proteins from the indicated cells and tissues, the RIPA lysis buffer (R0010, Solarbio, Beijing, China) was applied. A BCA kit (Pierce Biochemicals, USA) was applied to measure the total protein concentration of the samples. For western blot assay, an equal amount of total proteins (30–150 *μ*g) was loaded and separated using sodium dodecyl sulfate-polyacrylamide gel electrophoresis and subsequently transferred the resolved proteins from gel to polyvinylidene fluoride membranes. Following blocking using 5% fat-free milk, membranes were then incubated with one of METTL16 (CAT17676, 1 : 1,000, Cell Signaling Technology, USA), *β*-catenin (ab32572, 1 : 1,000, Abcam, UK), and GAPDH (ab181602, 1 : 1,000, Abcam, UK) primary antibodies overnight at 4°C. After being washed three times with phosphate-buffered saline with Tween 20, the membranes were subsequently incubated with a goat anti-rabbit HRP-conjugated secondary antibody (ab205718, 1 : 25,000, Abcam, UK) for 1 hr at room temperature. A chemiluminescent mixture (Millipore, Massachusetts, USA) was utilized to detect the amount of the target protein, followed by the utilization of ImageJ software to quantitate the grayscale values of the band of each target protein.

### 2.6. Cell Proliferation Assay

Cell Counting Kit-8 (CCK-8) (Beyotime Biotechnology, China), as well as 5-ethynyl-2′-deoxyuridine (EdU) (Beyotime Biotechnology, China), were used for the determination of cell proliferation. In summary, both control and transfected OC cells were plated in 96-well microplates at a concentration of 3,000 cells per well with 100 *μ*L of complete medium. Following incubation for 0, 24, 48, 72, and 96 hr, 10 *μ*L of CCK-8 solution was introduced to each well. CCK-8 is metabolized to produce a chromogen that is detected at 450 nm using a microplate reader. In the EdU assay, the aforementioned cells were plated in 96-well microplates at a concentration of 1 × 10^5^ per well, and 100 *μ*L of 50 *μ*M EdU medium was introduced to each well for a duration of 2 hr. After being washed with PBS, the cells were fixed for 30 min using 50 *μ*L of 4% paraformaldehyde. Following the PBS wash, 100 *μ*L of permeabilization solution (PBS solution with 0.5% Triton X-100) was introduced to each well and incubated on a destaining shaker for 10 min. Subsequently, in a dark environment, 50 *μ*L of click reaction solution (Beyotime Biotechnology, China) was added to each well and incubated for 30 min. The cell nuclei were then stained with Hoechst 33342 solution (Beyotime Biotechnology, China) for 10 min at room temperature while keeping them in the dark.

### 2.7. Transwell Assay

Cell invasion was measured with a transwell culture system. In summary, the matrigel (BD Biosciences, San Jose, CA, USA) was diluted with serum-free medium (1 : 9) and then applied to the inserts. The inserts were then placed in an incubator at 37°C for 2 hr until the matrigel was fully solidified. Subsequently, the number of 1 × 10^5^ suspended cells (200 *μ*L) was seeded in the upper chamber, while a chemoattractant of 10% FBS-containing culture medium (600 *μ*L) was added to the lower chamber. After a 12 hr incubation at 37°C, the cells that had invaded the bottom surface of the inserts were fixed with 4% paraformaldehyde for 20 min, stained with 0.1% crystal violet for 30 min, and counted using a light microscope.

### 2.8. Wound Healing Assay

Cells were cultured in 6-well plates and grown to 90% confluence at 37°C, and the cell monolayer was scraped using a 200 *μ*L micropipette tip to form a wound. Detached and damaged cells were washed off with PBS, and then cells were allowed to culture in a serum-free medium for 0–48 hr at 37°C. Wounds were observed and photographed at 0, 24, and 48 hr under an inverted microscope. The wound area at each time point in each group was quantified and compared to controls. The assays were performed in triplicate.

### 2.9. RNA Immunoprecipitation (RIP) Assay

A Magna RIP™ RNA-binding protein immunoprecipitation kit (Millipore, Massachusetts, USA) was used to perform the RIP assay. Briefly, the cells were lyzed using RIP lysis buffer to obtain RNA supernatants. After coupling of the beads with the anti-METTL16 antibody or negative control IgG at 4°C for 2 hr, the beads were added to the RNA supernatant and reacted at 4°C overnight. The relationships between target RNAs and genes were validated by RT-qPCR.

### 2.10. RNA Stability Assay

To evaluate the stability of lncRNA MALAT1, designated cells were cultured in six-well plates and treated with actinomycin-D (5 *μ*g/mL) for 0, 2, 4, 6, or 8 hr. Relative expression level of lncRNA MALAT1 was detected using RT-qPCR after total RNA extraction.

### 2.11. Cytoimmunofluorescence

Cells were seeded in 12-well plates in which glass coverslips were placed, and immunofluorescent staining was performed when cells grew to approximately 90% confluency. After 20 min fixation using 4% paraformaldehyde, the cell membrane was permeabilized with PBS solution containing 0.5% Triton X-100 (Beyotime Biotechnology, China) for 20 min, and after 2 hr blocking with 5% BSA, cells were incubated with one of the indicated primary antibodies at 4°C overnight in a humidified chamber. Then, following a triple wash, the cells were exposed to the Alexa Fluor 488 conjugated secondary antibody for 1 hr in a dark environment. The cell nuclei were then counterstained using Hoechst 33342 solution (Beyotime Biotechnology, China) for a duration of 10 min. Finally, images were visualized and captured using fluorescence microscopy.

### 2.12. Animal Experiment

Twelve BALB/c nude mice (female, 4 weeks old) were kept at the Animal Model Experimental Center of the First Affiliated Hospital of the Wannan Medical College. All the protocols of animal experiments were approved by the Animal Model Center of the Laboratory Animal Ethics Committee of the First Affiliated Hospital of the Wannan Medical College. The number of 5 × 10^6^ OVCAR3 cells was injected subcutaneously into the right flank of the mice and, tumor volumes were measured every 5 days. At the end of the experiment, mice were sacrificed, and the weights of excised tumors were recorded.

### 2.13. Statistical Analysis

SPSS 26.0 software (SPSS, USA) and GraphPad Prism 7.0 (GraphPad Software, USA) were used to statistically analyze the experimental data. Qualitative variables were compared between the high and low METTL16 groups using Fisher's exact test.  ^*∗*^*P* < 0.05,  ^*∗∗*^*P* < 0.01, and  ^*∗∗∗*^*P* < 0.001 were considered statistically significant.

## 3. Results

### 3.1. Differential Expression of METTL16 in EOC and Its Relationship with Clinicopathological Parameters

**Figure 1:**
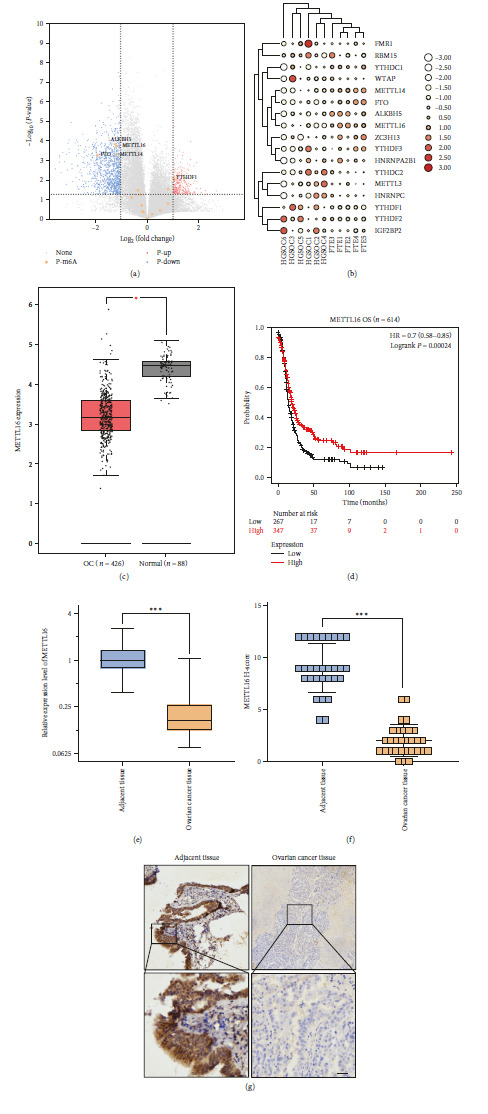
METTL16 is lowly expressed in EOC. (a, b) Volcano plot (a) and clustered heatmap (b) analyses of GSE190688 dataset revealed that METTL16 was differentially expressed in EOC. (c) The mRNA expression of METTL16 in normal ovary tissues (*n* = 88) and OC tissues (*n* = 426) in the TCGA database. (d) The overall survival (OS) EOC patients in the Kaplan–Meier plotter database with a low- or high-expression level of METTL16 were examined using the KM plotter online tool (http://www.kmplot.com). (e) The mRNA expression of METTL16 was detected by RT-qPCR in adjacent noncancerous tissues (*n* = 30) and EOC tissues (*n* = 30) of our own cohorts. (f) The *H*-score of METTL16 in adjacent noncancerous tissues (*n* = 30) and EOC tissues (*n* = 30) of our own cohorts. (g) The protein expression of METTL16 detected by IHC in adjacent noncancerous tissues (*n* = 30) and EOC tissues (*n* = 30) of our own cohorts (scale bar, 20 *μ*m).  ^*∗*^*P* < 0.05,  ^*∗∗*^*P* < 0.01, and  ^*∗∗∗*^*P* < 0.001. None: genes of no interest, P-m6A: m6A genes, P-up: upregulated genes, *P*-down: downregulated genes.

First, we screened the DEGs in EOC using the GSE190688 dataset and found that ALKBH5, METTL16, METTL14, FTO, and YTHDF1 were the DEGs in EOC among the m6A methylation-related regulators identified, while obviously decreased expression level of METTL16 was observed in EOC tissues of the GSE190688 dataset (Figures 1(a) and 1(b)). Next, the low expression level of METTL16 in EOC was also validated in the EOC datasets of the TCGA database (Figure 1(c)), and an obvious association of the low METTL16 expression level with the poor prognosis of EOC in the Kaplan–Meier database was also observed (Figure 1(d)). Subsequently, we assessed the expression level of METTL16 in EOC tissues and neighboring noncancerous tissues of our own validation cohorts using RT-qPCR and IHC techniques. The RT-qPCR and IHC results showed that in comparison to the adjacent noncancerous tissues, the EOC tissues exhibited obviously reduced METTL16 expression levels (Figures 1(e), 1(f), and 1(g)). IHC analysis demonstrated the presence of METTL16 within the nucleus and cytoplasm of OC cells (Figure 1(g)). Additionally, according to the IHC *H*-score of the relative protein expression level of METTL16 in 30 EOC tissues, the samples were categorized into low- and high-expression groups. Subsequently, we examined the associations between the expression level of METTL16 and various clinical outcomes, including age, FIGO stage, tumor size, tumor grade, and distant lymph node metastasis, in patients diagnosed with EOC (Table 1). According to the data presented in Table 1, we observed a significant correlation between METTL16 levels and each of the known adverse prognostic factors of EOC, such as FIGO stage, tumor size, and lymph node metastasis. These associations were consistent across different age groups and tumor grades.

### 3.2. Low METTL16 Expression Promotes Proliferation, Migration, and Invasion of EOC

**Figure 2:**
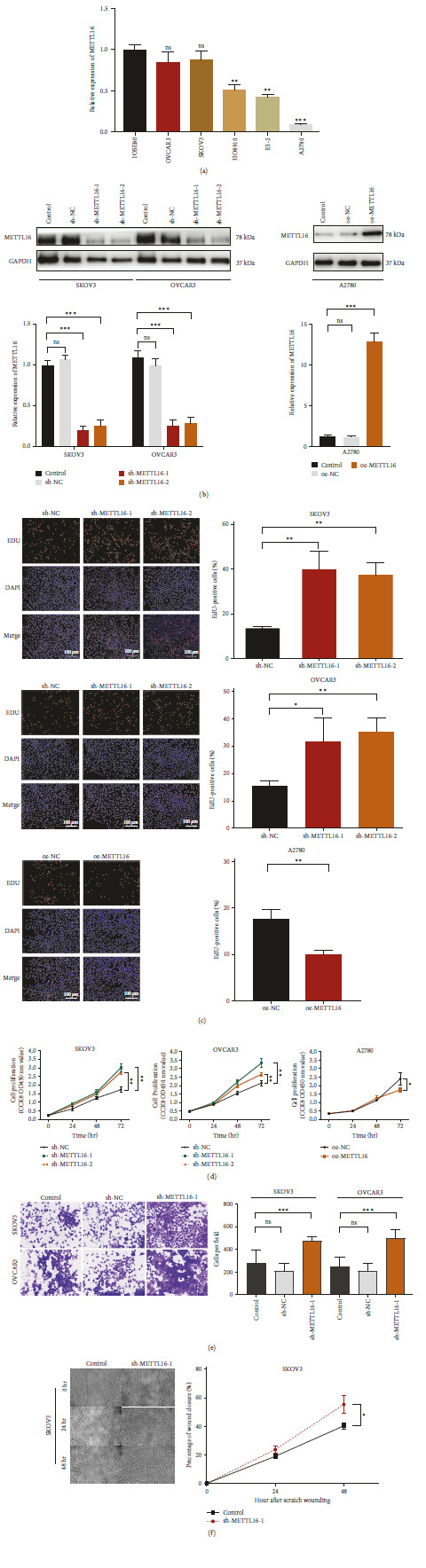
Effect of METTL16 on cell proliferation, migration, and invasion of OC cells. (a) Relative mRNA level of METTL16 in five OC cell lines (OVCAR3, SKOV3, HO8910, ES-2, and A2780), compared with human epithelial ovarian cell line (IOSE80). (b) Relative mRNA and protein expression levels of METTL16 72 hr after SKOV3 and OVCAR3 cells were transfected with sh-METTL16 (sh-METTL16-1 or sh-METTL16-2) or their control sh-RNA (sh-NC); relative mRNA and protein expression levels of METTL16 72 hr after A2780 cells were transfected with oe-METTL16 or oe-METTL16 control (oe-NC). (c, d) Detection of cell proliferation by EdU assay of SKOV3 and OVCAR3 cells after they were transfected with sh-METTL16 (sh-METTL16-1 or sh-METTL16-2) or their control sh-RNA (sh-NC) as well as A2780 cells after they were transfected with oe-METTL16 or oe-METTL16 control (oe-NC) (scale bar, 100 *μ*m). (e, f) Detection of invasion (e) and migration (f) of SKOV3 and OVCAR3 cells that were transfected with sh-METTL16 (sh-METTL16-1) or their control sh-RNA (sh-NC). ns: *P* > 0.05,  ^*∗*^*P* < 0.05,  ^*∗∗*^*P* < 0.01, and  ^*∗∗∗*^*P* < 0.001.

In order to choose suitable cell lines for functional experiments, we evaluated the expression levels of METTL16 in five OC cell lines (OVCAR3, SKOV3, HO8910, ES-2, and A2780) as well as the human epithelial ovarian cell line (IOSE80). Compared to the normal human epithelial ovarian cell line, the three OC cell lines exhibited significantly decreased mRNA expression levels of METTL16 (Figure 2(a)). Among these five OC cell lines, SKOV3 and OVCAR3 had a comparable, moderate mRNA expression level of METTL16, but A2780 had the lowest mRNA expression level of METTL16 (Figure 2(a)). Thus, three OC cell lines, SKOV3, OVCAR3, and A2780, were selected for our further study. First of all, we silenced the expression of METTL16 in SKOV3 and OVCAR3 OC cells (Figure 2(b)) and overexpressed METTL16 in A2780 cells (Figure 2(b)). The results from the EdU assay and CCK-8 assay indicated that the silencing of the METTL16 significantly promoted the cell proliferation rate of SKOV3 and OVCAR3 cells (Figures 2(c) and 2(d)), but the METTL16 overexpression in A2780 cells significantly reduced cell proliferation rate (Figures 2(c) and 2(d)). Furthermore, the observations from our transwell assay and scratch wound healing assay revealed that silencing of METTL16 markedly enhanced the invasion ability in SKOV3 and OVCAR3 cells (Figure 2(e)) and the migration ability in SKOV3 cells (Figure 2(f)), compared to their matched controls. These findings indicate that METTL16 may play a role of a tumor suppressor gene during the carcinogenesis of OC.

### 3.3. METTL16 Inhibits Tumor Growth in Nude Mice

**Figure 3:**
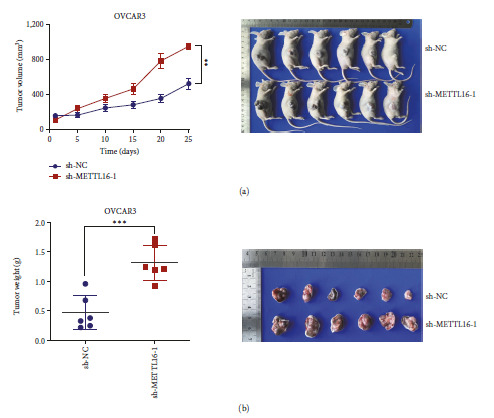
Effect of METTL16 on tumor growth in nude mice. (a) The growth curve of the volume of the subcutaneous tumor formed in two groups of nude mice that were implanted subcutaneously with OVCAR3 cells transfected with sh-NC or sh-METTL16-1. (b) The comparison of the weights of subcutaneous tumors dissected on day 25 from two groups of nude mice subcutaneously implanted with OVCAR3 cells transfected with sh-NC or sh-METTL16-1.  ^*∗*^*P* < 0.05,  ^*∗∗*^*P* < 0.01, and  ^*∗∗∗*^*P* < 0.001.

To investigate the role of METTL16 in the growth of EOC cells in vivo, the METTL16 deficient (sh-METTL16-1) and normal (sh-NC) SKOV3 cells were subcutaneously injected into the right flank of 12 nude mice (*n* = 6, each group). We discovered that METTL16 knockdown dramatically increased the tumor progression compared to that of control cells, as shown by increased tumor weight and volume (Figures 3(a) and 3(b)). These findings indicate that METTL16 may suppress tumor growth in vivo.

### 3.4. METTL16 Suppresses the Expression Level of lncRNA MALAT1

**Figure 4:**
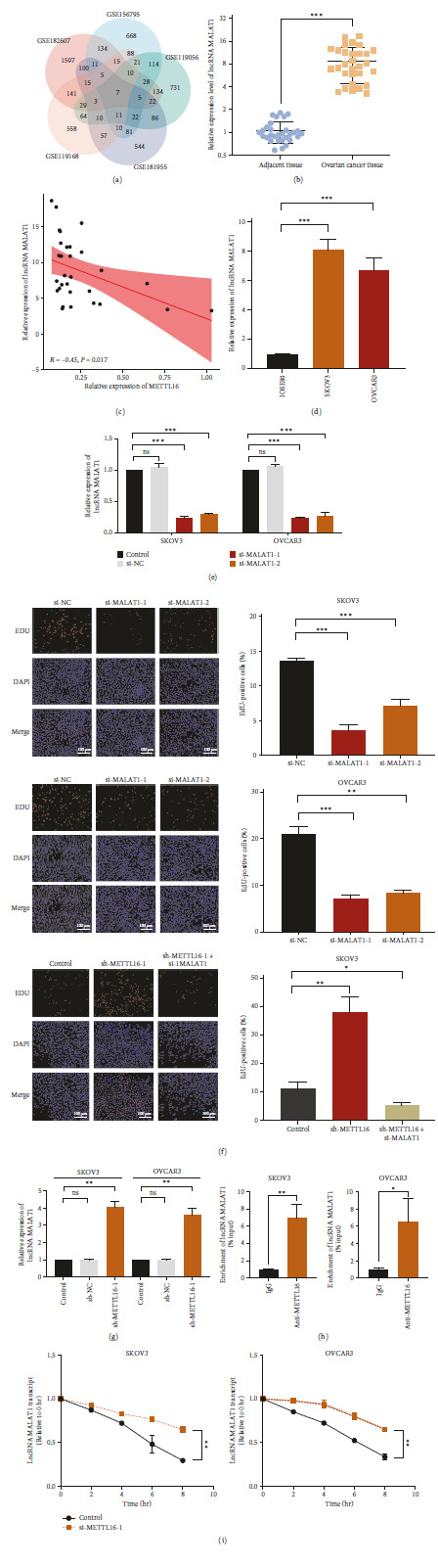
Expression level of lncRNA MALAT1 is downregulated by METTL16 in EOC cells. (a) The downstream target of METTL16, lncRNA MALAT1, was selected from the common seven genes identified from the five GEO datasets indicated. (b) The mRNA expression level of lncRNA MALAT1 was observed by RT-qPCR in adjacent noncancerous tissues (*n* = 30) and EOC tissues (*n* = 30). (c) The correlation between lncRNA MALAT1 and METTL16 expression levels in 30 EOC tissues was observed by RT-qPCR. *r* = −0.43, *P*=0.017 by Spearman correlation analysis. (d) Expression level of lncRNA MALAT1 in SKOV3 and OVCAR3 cells detected by RT-qPCR. (e) Relative mRNA expression level of MALAT1 72 hr after SKOV3 and OVCAR3 cells were transfected with si-MALAT1 (si-MALAT1-1 or si-MALAT1-2). (f) EdU assay was conducted to assess the proliferation ability of SKOV3 and OVCAR3 cells subsequent to the downregulation of lncRNA MALAT1 expression using si-MALAT1 (si-MALAT1-1 or si-MALAT1-2). Furthermore, the proliferation ability of SKOV3 cells was evaluated using the EdU assay following the knockdown of METTL16 alone, as well as the combined knockdown of METTL16 and lncRNA MALAT1 (scale bar, 100 *μ*m). (g) LncRNA MALAT1 expression level detected by RT-qPCR in SKOV3 and OVCAR3 cells with METTL16 stable knockdown by sh-METTL16-1. (h) RIP assay was performed in SKOV3 and OVCAR3 cells, followed by RT-qPCR to detect enrichment of lncRNA MALAT1. (i) RT-qPCR analysis of lncRNA MALAT1 in METTL16-transfected SKOV3 and OVCAR3 cells treated with actinomycin D (5 *μ*g/mL) for 0, 2, 4, 6, or 8 hr. ns: *P* > 0.05,  ^*∗*^*P* < 0.05,  ^*∗∗*^*P* < 0.01, and  ^*∗∗∗*^*P* < 0.001. EdU, 5-ethynyl-2′-deoxyuridine; RIP, RNA immunoprecipitation.

To find out the potential mechanisms underlying the oncogenic effect of downregulated METTL16 in EOC, the downstream genes that are aberrantly expressed in EOC and regulated by METTL16 were analyzed using datasets from five GEO datasets of EOC (GSE182607, GSE156795, GSE119056, GSE119168, and GSE181955). A differentially expressed lncRNA, MALAT1, was identified from these five GEO datasets (Figure 4(a)). Subsequently, we assessed the expression profile of lncRNA MALAT1 in 30 EOC tissues and adjacent noncancerous tissues from our independent validation cohorts using RT-qPCR. The results revealed a significant elevation in the expression of lncRNA MALAT1 in EOC tissues (Figure 4(b)). LncRNA MALAT1 expression level was inversely correlated with METTL16 expression level in these 30 EOC tissues (Figure 4(c)). In addition, the expression level of lncRNA MALAT1 was significantly increased in SKOV3 and OVCAR3 cells (Figure 4(d)). To investigate the impact of lncRNA MALAT1 on OC cells, we utilized si-RNA to downregulate the expression level of lncRNA MALAT1 in SKOV3 and OVCAR3 cells (Figure 4(e)). Subsequently, we conducted the EdU assays. The results demonstrated that the knockdown of lncRNA MALAT1 significantly suppressed cell proliferation rates in SKOV3 and OVCAR3 cells (Figure 4(f)). Additionally, the EdU assay indicated that the proliferation ability of SKOV3 cells was enhanced following the downregulation of METTL16 by sh-RNA but attenuated after the downregulation of lncRNA MALAT1 by si-RNA (Figure 4(f)). Then, the modulation of the lncRNA MALAT1 by METTL16 was evaluated using RT-qPCR. The results showed that the expression level of lncRNA MALAT1 was increased after METTL16 knockdown in SKOV3 and OVCAR3 cells (Figure 4(g)), suggesting that METTL16 may regulate MALAT1 by an interaction between them. Next, we examined whether METTL16 may regulate MALAT1 via an intermolecular interaction by RIP-qPCR using METTL16 antibody. The RIP-qPCR data show that enrichment of MALAT1 by METTL16 antibody, demonstrating that there exists an interaction between these two molecules and METTL16 may directly regulate MALAT1 (Figure 4(h)). Therefore, we further evaluated the expression level of lncRNA MALAT1 in SKOV3 and OVCAR3 cells transfected with siMETTL16 by RT-qPCR after blocking the de novo synthesis of lncRNA MALAT1 using actinomycin D. The data showed that the silencing of METTL16 delayed the degradation of lncRNA MALAT1 (Figure 4(i)). Thus, these findings indicate that METTL16 binds to lncRNA MALAT1 and decreases its stability, thereby inhibiting the proliferative capacity of OC cells.

### 3.5. METTL16 Inhibits the Malignant Progression of EOC through the lncRNA MALAT1/*β*-Catenin Axis

**Figure 5:**
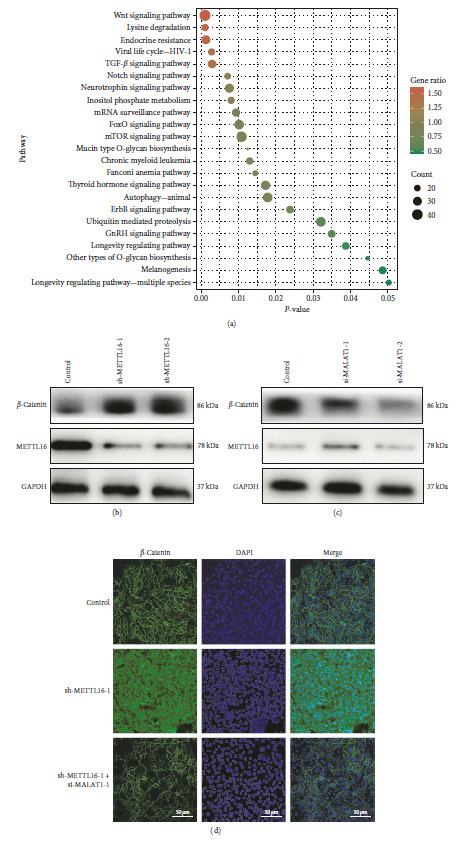
METTL16 suppresses EOC progression through the lncRNA MALAT1/*β*-catenin axis. (a) KEGG enrichment analysis of genes associated with METTL16 and lncRNA MALAT1. (b) Western blotting assay was conducted to determine the protein expression level of *β*-catenin in SKOV3 cells following the knockdown of METTL16. (c) Western blotting assay was performed to assess the *β*-catenin expression level in SKOV3 cells subsequent to the combined knockdown of both METTL16 and lncRNA MALAT1. (d) cytoimmunofluorescence staining assay was employed to assess the protein expression level of *β*-catenin following the knockdown of METTL16 alone or combined knockdown of both METTL16 and lncRNA MALAT1 (scale bar, 50 *μ*m).

To further explore the possible mechanism underlying the promoting effects of downregulated METTL16 and upregulated lncRNA MALAT1 on the malignant progression of EOC, the Kyoto Encyclopedia of Genes and Genomes (KEGG) pathways were investigated for these genes that were significantly associated with the expression of METTL16 and lncRNA MALAT1 on the bioinformatic analysis website, DAVID (the Database for Annotation, Visualization and Integrated Discovery). The results of KEGG suggested that the majority of the genes that were significantly associated with the expression of METTL16 and lncRNA MALAT1 were enriched in the Wnt/*β*-catenin pathway (Figure 5(a)), clarifying that METTL16 possibly plays its tumor suppressive role through inhibiting the Wnt/*β*-catenin pathway. Furthermore, Western blotting assay verified *β*-catenin upregulation after METTL16 knockdown in SKOV3 cells (Figure 5(b)). Significantly decreased *β*-catenin expression level was also observed after lncRNA MALAT1 was also downregulated by si-RNA in METTL16-deficient SKOV3 cells (Figure 5(c)). Additionally, in cytoimmunofluorescence staining experiments, we found that loss of METTL16 prompted more *β*-catenin protein to be translocated to the cell nucleus, whereas this phenomenon disappeared after lncRNA MALAT1 was downregulated by si-RNA (Figure 5(d)). In light of the above results, we demonstrate that METTL16 can ultimately inhibit the malignant progression of EOC cells by promoting the degradation of lncRNA MALAT1, which in turn leads to *β*-catenin downregulation and reduced intranuclear translocation of *β*-catenin.

## 4. Discussion

OC is a prevalent malignancy of the female reproductive system, and it holds the highest mortality rate among gynecological tumors. Apart from the challenge of early detection, the unfavorable prognosis of patients is also attributed to the metastasis and recurrence of tumors, which has become a prominent concern in the medical field. Therefore, a deep appreciation of the pathogenesis of OC is mandatory. RNA epigenetic modifications play a pivotal role in the malignant progression of OC. Therefore, further exploration of genes involved in epigenetic modifications in OC and identification of potential therapeutic targets will provide a valuable basis for effective improvement of the prognosis of OC. In this study, we observed that METTL16 is differentially expressed in EOC and is associated with the prognosis of EOC by bioinformatic analysis of the publicly available datasets of EOC from TCGA. We validated METTL16 expression level and this association with the clinicopathologic parameters of EOC patients of our own cohorts. We examined clinical tissue samples from patients with EOC by IHC and RT-qPCR and clarified that METTL16 mRNA and protein expression levels were obviously decreased compared to neighboring noncancerous tissues. Subsequently, we performed a statistical analysis of the METTL16 protein expression using IHC and evaluated the association between the quantified IHC *H*-score with the clinicopathological features of EOC patients. The outcomes showed that the low-expression level of METTL16 was obviously linked with poor prognostic factors, such as FIGO stage, tumor size, and lymph node metastasis. We explored the effect of low METTL16 expression on the biological behavior of EOC cells after we validated that METTL16 is differentially expressed in EOC and is associated with the prognosis of EOC. The findings of in vitro functional assays demonstrated that reduced METTL16 expression levels significantly enhanced OC cell proliferation, migration, and invasion. At the same time, functional assays in vivo also observed that low levels of METTL16 expression promoted tumor growth in EOC xenografts in mice. Based on the data from both in vitro and in vivo experiments, we demonstrate that METTL16 may be a crucial factor in the malignant development of EOC.

A unique nuclear expression element is contained in the 3′ end of lncRNA MALAT1, which could protect MALAT1 from degradation and maintain its oncogenic activity in a variety of cancers due to its three-helix structure [20–22]. Recently, many studies have shown that alteration of the m6A methylation modification of the lncRNA MALAT1 affects tumor progression. For example, METTL3 potentiates the stability of MALAT1 by m6A modification with the assistance of an RNA-binding protein, HuR, thereby facilitating the malignant progression in gliomas with IDH wild-type [23]. Overexpression of the lncRNA MALAT1 has been shown to promote the development of inflammation, as well as proliferation and invasion of tumor cells in the tumor microenvironment of EOC, ultimately resulting in the development of cancer [24]. Although it has been shown that METTL16 can bind to the 3′-terminal triple helix of lncRNA MALAT1 [12], its specific function after binding is not yet clear. Our study found that the downregulated expression of METTL16 enhances the oncogenic effect of lncRNA MALAT1 in OC cells, and the upregulation of lncRNA MALAT1 promotes the proliferation of OC cells. We then observed a significant negative association between the expression levels of METTL16 and lncRNA MALAT1 in EOC tissues. Based on the above experimental results, we further analyzed the correlation between METTL16 and MALAT1 by performing RIP and RNA stability experiments. The results of our study showed that METTL16 inhibited its oncogenic activity in EOC by binding lncRNA MALAT1 to promote its degradation, which is a novel finding. However, whether METTL16 regulates the degradation of lncRNA MALAT1 through m6A methylation modification needs to be further investigated.

The Wnt/*β*-catenin pathway is a canonical pathway of the Wnt signaling pathway in the physiological settings. Upon activation, this pathway results in a cascade of hallmark events—the buildup of *β*-catenin in the cytosol, the translocation of *β*-catenin to the cell nucleus, and the binding of translocated *β*-catenin with T-cell transcription factor/lymphoid enhancer factor that ultimately activates expression of downstream target genes [25]. In the pathophysiological settings, the Wnt/*β*-catenin signaling pathway plays a crucial role in the progression of cancer by acting as a significant regulator of the various biological processes. Persistent activation of the Wnt pathway accompanied by aberrant expression of *β*-catenin protein is present in approximately 40% of EOC [26]. In our study, we demonstrated that METTL16 coregulates the expression of *β*-catenin protein with lncRNA MALAT1. This was mainly characterized by an increase in the amount of *β*-catenin protein after the downregulation of METTL16, whereas the amount of *β*-catenin protein decreased upon the downregulation of lncRNA MALAT1. Guo et al. [27] reported that the reduction of MALAT1 decreased *β*-catenin expression in EOC, which is consistent with our findings. Additionally, we demonstrated that METTL16 was involved in the nuclear migration of *β*-catenin protein and facilitated the transfer of *β*-catenin protein from OC cell membranes to the cell nucleus. The *β*-catenin protein that is accumulated within the cell nucleus further contributes to the malignant progression of EOC. Interestingly, the nuclear translocation phenomenon of *β*-catenin protein was reversed when we downregulated lncRNA MALAT1. In conclusion, based on the aforementioned experimental results, we determined that METTL16 played a pivotal role in the formation of OC, acting as a tumor suppressor via the lncRNA MALAT1/*β*-catenin axis. Therefore, METTL16 is expected to be a potential therapeutic target for OC.

## 5. Conclusions

In conclusion, our study demonstrates that METTL16 is significantly downregulated in EOC, which has a correlation with the known adverse prognostic factors of EOC. Mechanistically, METTL16 is able to inhibit the development of EOC by binding and promoting the degradation of lncRNA MALAT1 and thus downregulating *β*-catenin expression.

## Figures and Tables

**Table 1:** Correlation between clinicopathologic parameters and expression level of METTL16.

Clinicopathological parameters	METTL16 expression level		
	High (4)	Low (26)	*P*-value
Age (years)			
<50	3	14	
≥50	1	12	0.6129
FIGO stage			
Ⅰ + Ⅱ	4	4	
Ⅲ + Ⅳ	0	22	0.0026^*∗∗*^
Tumor size (cm)			
<5	4	3	
≥5	0	23	0.0013^*∗∗*^
Lymph node metastasis			
Yes	1	23	
No	3	3	0.0181^*∗*^
Grade			
G1	2	7	
G2 + G3	2	19	0.5632

^*∗*^*P* < 0.05;  ^*∗∗*^*P* < 0.01.

## Data Availability

The data utilized to substantiate the discoveries of this investigation are incorporated within the article. The datasets employed and/or examined during the present study can be obtained from the corresponding author upon a reasonable inquiry.

## References

[1] Stewart C., Ralyea C., Lockwood S. (2019). Ovarian cancer: an integrated review. *Seminars in Oncology Nursing*.

[2] Conte1 C., Fagotti1 A., Avesani G. (2021). Update on the secondary cytoreduction in platinum-sensitive recurrent ovarian cancer: a narrative review. *Annals of Translational Medicine*.

[3] Torre L. A., Trabert B., DeSantis C. E. (2018). Ovarian cancer statistics, 2018. *CA: A Cancer Journal for Clinicians*.

[4] Huang H., Weng H., Chen J. (2020). m^6^A modification in coding and non-coding RNAs: roles and therapeutic implications in cancer. *Cancer Cell*.

[5] Zhang C., Liu J. H., Guo H. (2021). m6A RNA methylation regulators were associated with the malignancy and prognosis of ovarian cancer. *Bioengineered*.

[6] Nie S., Zhang L., Liu J. (2021). ALKBH5-HOXA10 loop-mediated JAK2 m6A demethylation and cisplatin resistance in epithelial ovarian cancer. *Journal of Experimental & Clinical Cancer Research*.

[7] Wang J., Xu J., Li K. (2020). Identification of WTAP-related genes by weighted gene co-expression network analysis in ovarian cancer. *Journal of Ovarian Research*.

[8] Li Q., Ren C.-C., Chen Y.-N. (2021). A risk score model incorporating three m6A RNA methylation regulators and a related network of miRNAs-m6A regulators-m6A target genes to predict the prognosis of patients with ovarian cancer. *Frontiers in Cell and Developmental Biology*.

[9] Bi X., Lv X., Liu D. (2021). METTL3 promotes the initiation and metastasis of ovarian cancer by inhibiting CCNG2 expression via promoting the maturation of pri-microRNA-1246. *Cell Death Discovery*.

[10] Choe J., Lin S., Zhang W. (2018). mRNA circularization by METTL3–eIF3h enhances translation and promotes oncogenesis. *Nature*.

[11] Weng H., Huang H., Wu H. (2018). METTL14 inhibits hematopoietic stem/progenitor differentiation and promotes leukemogenesis via mRNA m^6^A modification. *Cell Stem Cell*.

[12] Brown J. A., Kinzig C. G., DeGregorio S. J., Steitz J. A. (2016). Methyltransferase-like protein 16 binds the 3′-terminal triple helix of MALAT1 long noncoding RNA. *Proceedings of the National Academy of Sciences*.

[13] Warda A. S., Kretschmer J., Hackert P. (2017). Human METTL16 is a *N*^6^-methyladenosine (m^6^A) methyltransferase that targets pre-mRNAs and various non-coding RNAs. *EMBO Reports*.

[14] Pendleton K. E., Chen B., Liu K. (2017). The U6 snRNA m^6^A methyltransferase METTL16 regulates SAM synthetase intron retention. *Cell*.

[15] Mendel M., Chen K.-M., Homolka D. (2018). Methylation of structured RNA by the m^6^A writer METTL16 Is essential for mouse embryonic development. *Molecular Cell*.

[16] Giannakis M., Mu X. J., Shukla S. A. (2016). Genomic correlates of immune-cell infiltrates in colorectal carcinoma. *Cell Reports*.

[17] Hou M., Guo X., Chen Y., Cong L., Pan C. (2020). A prognostic molecular signature of *N*^6^-methyladenosine methylation regulators for soft-tissue sarcoma from the cancer genome Atlas database. *Medical Science Monitor*.

[18] Li K., Luo H., Luo H., Zhu X. (2020). Clinical and prognostic pan-cancer analysis of m6A RNA methylation regulators in four types of endocrine system tumors. *Aging*.

[19] Wang P., Wang X., Zheng L., Zhuang C. (2020). Gene signatures and prognostic values of m6a regulators in hepatocellular carcinoma. *Frontiers in Genetics*.

[20] Torabi S.-F., DeGregorio S. J., Steitz J. A. (2021). tRNA-like leader-trailer interaction promotes 3′-end maturation of MALAT1. *RNA*.

[21] Yonkunas M. J., Baird N. J. (2019). A highly ordered, nonprotective MALAT1 ENE structure is adopted prior to triplex formation. *RNA*.

[22] Goyal B., Yadav S. R. M., Awasthee N., Gupta S., Kunnumakkara A. B., Gupta S. C. (2021). Diagnostic, prognostic, and therapeutic significance of long non-coding RNA MALAT1 in cancer. *Biochimica et Biophysica Acta (BBA) - Reviews on Cancer*.

[23] Chang Y.-Z., Chai R.-C., Pang B. (2021). METTL3 enhances the stability of MALAT1 with the assistance of HuR via m6A modification and activates NF-*κ*B to promote the malignant progression of IDH-wildtype glioma. *Cancer Letters*.

[24] Mao T.-L., Fan M.-H., Dlamini N., Liu C.-L. (2021). LncRNA MALAT1 facilitates ovarian cancer progression through promoting chemoresistance and invasiveness in the tumor microenvironment. *International Journal of Molecular Sciences*.

[25] Zhou L., Jiang J., Huang Z. (2022). Hypoxia-induced lncRNA *STEAP3-AS1* activates Wnt/*β*-catenin signaling to promote colorectal cancer progression by preventing m^6^A-mediated degradation of *STEAP3* mRNA. *Molecular Cancer*.

[26] Zyla R. E., Olkhov-Mitsel E., Amemiya Y. (2021). *CTNNB1* mutations and aberrant *β*-catenin expression in ovarian endometrioid carcinoma: correlation with patient outcome. *American Journal of Surgical Pathology*.

[27] Guo C., Wang X., Chen L.-P. (2018). Long non-coding RNA MALAT1 regulates ovarian cancer cell proliferation, migration and apoptosis through Wnt/*β*-catenin signaling pathway. *European Review for Medical and Pharmacological Sciences*.

